# Validation of questionnaire-reported chest wall abnormalities with a telephone interview in Swiss childhood cancer survivors

**DOI:** 10.1186/s12885-021-08425-z

**Published:** 2021-07-08

**Authors:** Rahel Kasteler, Christa Lichtensteiger, Christina Schindera, Marc Ansari, Claudia E. Kuehni, for the Swiss Pediatric Oncology Group (SPOG) Scientific Committee, J. Rössler, M. Ansari, M. Beck Popovic, P. Brazzola, J. Greiner, F. Niggli, H. Hengartner, C. Kuehni, F. Schilling, K. Scheinemann, N. von der Weid, N. Gerber

**Affiliations:** 1 Childhood Cancer Registry, Institute of Social and Preventive Medicine, University of Bern, Mittelstrasse 43, 3012 Bern, Switzerland; 2 Department of Pediatrics, Inselspital, Bern University Hospital, University of Bern, 3010 Bern, Switzerland; 3 Division of Pediatric Hematology/Oncology, University Children’s Hospital Basel, 4056 Basel, Switzerland; 4 Department of Women, Child and Adolescents, Pediatric Oncology and Hematology Unit, Geneva University Hospital, 1205 Geneva, Switzerland; 5 Platform of Pediatric Onco-Hematology research (CANSEARCH Research Laboratory), Department of Pediatrics, Gynecology, and Obstetrics, University of Geneva, 1205 Geneva, Switzerland

**Keywords:** Chest wall deformity, Swiss Childhood Cancer Survivor Study, Late effects, Cancer treatment

## Abstract

**Background:**

Chest wall abnormalities are a poorly studied complication after treatment for childhood cancer. Chest wall abnormalities are not well-described in the literature, and little is known on the impact on daily life of survivors.

**Methods:**

We investigated prevalence and risk factors of chest wall abnormalities in childhood cancer survivors in a nationwide, population-based cohort study (Swiss Childhood Cancer Survivor Study) with a questionnaire survey. We then interviewed a nested sample of survivors to validate types of chest wall abnormalities and understand their impact on the daily life of survivors.

**Results:**

Forty-eight of 2382 (95%CI 2–3%) survivors reported a chest wall abnormality. Risk factors were older age at cancer diagnosis (16–20 years; OR 2.5, 95%CI 1.0–6.1), lymphoma (OR 3.8, 95%CI 1.2–11.4), and central nervous system tumors (OR 9.5, 95%CI 3.0–30.1) as underlying disease, and treatment with thoracic radiotherapy (OR 2.0, 95%CI 1.0–4.2), surgery to the chest (OR 4.5, 95%CI 1.8–11.5), or chemotherapy (OR 2.9, 95%CI 1.0–8.1). The nature of the chest wall abnormalities varied and included thoracic wall deformities (30%), deformations of the spine (5%) or both (55%), and scars (10%). Chest wall abnormalities affected daily life in two thirds (13/20) of those who reported these problems and necessitated medical attention for 15 (75%) survivors.

**Conclusion:**

It is important that, during follow-up care, physicians pay attention to chest wall abnormalities, which are rare late effects of cancer treatment, but can considerably affect the well-being of cancer survivors.

**Supplementary Information:**

The online version contains supplementary material available at 10.1186/s12885-021-08425-z.

## Background

Chest wall abnormalities have been reported in a widely variable proportion of childhood cancer survivors. Available studies were small, including 16 to 143 participants [1–9], or focused on patients with selected cancer diagnoses only, e.g., chest wall sarcoma [1], central nervous system (CNS) tumours [10], neuroblastoma [2, 9], or Wilms tumour [3, 4]. Some studies reported on specific cancer treatments such as thoracic radiotherapy, radiotherapy to the spine [6], abdominal radiotherapy [7, 8], or surgical interventions for solid tumours [5]. Such studies are not representative of the entire population of childhood cancer survivors. In large, cross-sectional studies, 1.4% [11] (North America, multicenter study) to 2.0% [12] (Switzerland, population-based study) of survivors reported chest wall abnormalities in questionnaire surveys [10–12]. Those investigations used one single question on chest wall abnormalities, which did not enable understanding of the type of chest wall abnormalities as no exact definition was attached to the question. Survivors might not understand the term “chest wall abnormalities” in the way researchers intend. They might choose to report different health issues as chest wall abnormalities, including thoracic wall deformities, spinal deformities, breast asymmetries, or scars. No study investigated whether and how chest wall abnormalities affect the daily life of survivors and if medical care is needed.

With this study, we wanted to expand the epidemiological knowledge on chest wall abnormalities in survivors. First, we assessed the prevalence of chest wall abnormalities reported by survivors in Switzerland and investigated cancer and cancer-treatment-related risk factors for chest wall abnormalities. Second, we interviewed a nested sample of survivors to validate and clarify the type of chest wall abnormalities reported by survivors and their impact on daily life. Last, we conducted a systematic review of the available literature on chest wall abnormalities in childhood cancer survivors.

## Methods

### Swiss childhood Cancer survivor study

The Swiss Childhood Cancer Survivor Study (SCCSS) is a population-based, long-term follow-up study of patients registered in the Swiss Childhood Cancer Registry (SCCR, www.childhoodcancerregistry.ch). Participants have been diagnosed with leukemia, lymphoma, CNS tumors, malignant solid tumors, or Langerhans cell histiocytosis after 1976 and before the age of 21 years. Participants, who have survived ≥5 years since initial cancer diagnosis and were alive at the time of the study, received a questionnaire between 2007 and 2013. Nonresponders received a second copy of the questionnaire four to 6 weeks later. If they again did not answer, we contacted them by phone. In total 2382 survivors replied (Supplementary figure S1). Detailed methods of the SCCSS have been published [12–14].

### Outcome: chest wall abnormalities

The SCCSS questionnaire, like the North Amercian [15] and British [16] Childhood Cancer Survivor Studies, includes one question on chest wall abnormalities in the section on pulmonary health: *“Have you ever been told by a doctor that you have or have had changes to your thorax and/or ribs?”* and possible answers included *ever in life* *(yes/no)*, *since when (year)*, and *currently* *(yes/no)* (Supplementary figure S2).

### Validation of outcome by telephone interview

In a nested follow-up study, we sent a letter to all survivors who had reported a chest wall abnormality in the questionnaire to invite them to take part in a telephone interview. All those were at least 18 years old, still alive, had consented to participation in further studies, and had a valid telephone number. Survivors were contacted by telephone between July 2017 and September 2017 by one investigator (CL) (Supplementary figure S1). The purpose of the structured interview was to determine the medical problems underlying the reported chest wall abnormalities. We sought information on 1) deformations of the chest wall that included asymmetric chest wall, pectus excavatum, pectus carinatum, completely or partially missing ribs, deformation of the breast, muscular abnormalities, or other deformations of the chest wall or ribs; 2) deformations of the spine including scoliosis, hyperkyphosis, hyperlordosis, or other deformations of the spine; 3) scars on the chest wall. We also asked about the impact of chest wall abnormalities on daily life which could include general complaints as well as cosmetic problems, respiration problems, flexibility impairments, pain because of the chest wall abnormalities, and impairment in activities of daily living. Finally, we asked whether medical attention—consultation with a physician, diagnostic investigations, operations, and physiotherapy—had been sought. The questionnaire used for the interview is available as supplementary material in its original form in German and as an English translation.

The Ethics Committee of the Canton of Bern approved the SCCR and the SCCSS (KEK-BE: 166/2014), and the Swiss Childhood Cancer Survivor Study is registered at ClinicalTrials.gov (identifier: NCT03297034).

### Covariates: demographic and cancer-related characteristics

We obtained cancer characteristics from the SCCR including age at diagnosis, year of diagnosis, cancer diagnosis according to the International Classification of Childhood Cancer, 3rd edition [17], and details on radiotherapy, surgery, and chemotherapy. We combined total body irradiation, mantle field radiation, and radiation to the thorax, lungs, mediastinum, or thoracic spine to thoracic radiotherapy (yes/no). Surgery to the chest (yes/no) included the clavicles, scapulae and ribs, tumor excision from soft tissue on thorax, thoracic muscles, thoracic spine, and tumor or lymph node biopsies on the chest wall.

### Statistical analysis

We reported prevalence of chest wall abnormalities overall and stratified by sex, age at cancer diagnosis (0–5; 6–10; 11–15; 16–20), years of diagnosis (1976–1990; 1991–2005), cancer diagnosis (leukemia; lymphoma; CNS tumor; other tumors), and cancer treatment (thoracic radiotherapy (yes, no); surgery to the chest (yes, no); any chemotherapy (yes, no)). We identified demographic and cancer-related risk factors for chest wall abnormalities using univariable and multivariable logistic regression. All analyses were done in Stata (Version 14; Stata Corporation, Austin, TX).

### Systematic literature review

We conducted a literature review searching for relevant articles in the two bibliographic databases PubMed and Embase Ovid, last updated December 31st 2019. Both databases were searched using thesaurus terms (MeSH, Emtree) and textwords. We applied restrictions to language and searched studies on humans only, and excluded from the search conference abstracts, letters to the editor, and editorials. To retrieve further relevant publications, we checked the reference lists of studies included and added Google scholar for a full-text search. An information specialist from the University Library of Bern was consulted to set up the search strategies in order to ensure optimal data acquisition.

The search results were screened in two steps by two independent reviewers (RK, CL) and assessed according to relevance and eligibility criteria (PRISMA flow diagram). We excluded articles on deformations of the spine (kyphosis, kyphoscoliosis, hyperlordosis, and scoliosis), as this was not the main focus of this project. Additional articles were searched by screening the reference list of suitable systematic reviews found in the two databases. For details on search strategies and search platforms, see Supplementary Text and Supplementary figure S3.

## Results

### Prevalence and risk factors of chest wall abnormalities

Among the 2382 survivors who participated in the SCCSS, 2% (48/2, 382) reported a chest wall abnormality (95% confidence interval [95%CI] 1.5–2.7). Male survivors were more often affected (2.5, 95%CI 1.8–3.5) than females (1.4, 95%CI 0.8–2.3) (Table 1). Median age at study was 31 years (interquartile range [IQR] 25–38) and survivors who were older at cancer diagnosis (16–20 years, 4.2, 95%CI 2.2–7.9) had a higher prevalence of chest wall abnormalities compared to those who were younger. Prevalence did not change over time, being 2 and 2.1% in the periods of 1976–1990 and 1991–2005. When comparing underlying diagnoses, chest wall abnormalities were most frequently reported by survivors of lymphoma (3.7, 95%CI 2.3–6.0) and CNS tumors (3.8, 95%CI 2.2–6.4), but rarely by participants treated for leukemia (0.6, 95%CI 0.2–1.5). Nearly 8% of survivors treated with surgery to the chest and 6% treated with thoracic radiotherapy reported a chest wall abnormality.

**Table 1:** Characteristics of Swiss childhood cancer survivors overall and of those reporting chest wall abnormalities

	Overall *N* = 2382 (100%)		Survivors reporting chest wall abnormalities *N* = 48 (2.0, 95%CI 1.5–2.7)		
	n	%^a^	n	%^b^	(95%CI)
Sex					
Female	1111	46.6%	16	1.4%	(0.8–2.3)
Male	1271	53.4%	32	2.5%	(1.8–3.5)
Age at diagnosis (years)					
0–5	1108	46.5%	20	1.8%	(1.2–2.8)
6–10	521	21.9%	7	1.3%	(0.6–2.8)
11–15	540	22.7%	12	2.2%	(1.3–3.9)
16–20	213	8.9%	9	4.2%	(2.2–7.9)
Median (IQR) age at study (years)	31.1	(24.6–38.2)	32.1	(25.8–38.1)	
Year of diagnosis					
1976–1990	845	35.5%	18	2.1%	(1.3–3.4)
1991–2005	1537	64.5%	30	2.0%	(1.4–2.8)
Diagnosis					
I: Leukemia	773	32.5%	5	0.6%	(0.2–1.5)
II: Lymphoma	428	17.9%	16	3.7%	(2.3–6.0)
III: CNS tumor	345	14.5%	13	3.8%	(2.2–6.4)
IV–XII: Other tumors	836	35.1%	14	1.7%	(1.0–2.8)
Thoracic radiotherapy ^c^					
No	2075	87.1%	31	1.5%	(1.1–2.1)
Yes	307	12.9%	17	5.5%	(3.5–8.7)
Surgery to the chest ^d^					
No	2290	96.1%	41	1.8%	(1.3–2.4)
Yes	92	3.9%	7	7.6%	(3.7–15.2)
Any chemotherapy					
No	414	17.4%	6	1.4%	(0.7–3.2)
Yes	1968	82.6%	42	2.1%	(1.6–2.9)

*Abbreviations*: *CI* Confidence interval, *N* Number

^a^ Column percentages are given

^b^ Row percentages are given

^c^ Including the following radiation fields: total body irradiation, mantle field, thorax, lungs, mediastinum, or thoracic spine

^d^ Including surgery to clavicle, scapulae and ribs, tumor excision from soft tissue on thorax, muscles on thorax, spine of thorax, and tumor or lymph node biopsy on the chest wall

In a multivariable regression, the following factors remained independently associated with chest wall abnormalities: male sex (odds ratio [OR] 1.8, 95%CI 1.0–3.3), older age at cancer diagnosis (OR 2.5, 95%CI 1.0–6.1), lymphoma (OR 3.8, 95%CI 1.2–11.4), CNS tumor (OR 9.5, 95%CI 3.0–30.1), thoracic radiotherapy (OR 2.0, 95%CI 1.0–4.2), surgery to the chest (OR 4.5, 95%CI 1.8–11.5), and chemotherapy (OR 2.9, 95%CI 1.0–8.1) (Table 2).

**Table 2:** Demographic and cancer-related risk factors for chest wall abnormalities in Swiss childhood cancer survivors

Total *N* = 2382	Chest wall abnormalities (*n* = 48)					
	OR_crude_^a^	(95%CI)	P^**b**^	OR_adj_^c^	(95%CI)	P^**c**^
Sex			**0.059**			**0.062**
Female	Ref.			Ref.		
Male	1.8	(1.0–3.2)		1.8	(1.0–3.3)	
Age at diagnosis (years)			**0.125**			**0.017**
0–5	Ref.			Ref.		
6–10	0.7	(0.3–1.8)		0.5	(0.2–1.2)	
11–15	1.2	(0.6–2.5)		0.7	(0.3–1.6)	
16–20	2.4	(1.1–5.3)		2.5	(1.0–6.1)	
Year of diagnosis			**0.768**			**0.602**
1976–1990	Ref.			Ref.		
1991–2005	0.9	(0.5–1.7)		0.8	(0.5–1.6)	
Diagnosis			**< 0.001**			**< 0.001**
I: Leukemia	Ref.			Ref.		
II: Lymphoma	6.0	(2.2–16.4)		3.8	(1.2–11.4)	
III: CNS tumor	6.0	(2.1–17.0)		9.5	(3.0–30.1)	
IV–XII: Other tumors	2.6	(0.9–7.3)		2.1	(0.7–6.1)	
Thoracic radiotherapy ^d^			**< 0.001**			**0.058**
No	Ref.			Ref.		
Yes	3.9	(2.1–7.1)		2.0	(1.0–4.2)	
Surgery to the chest ^e^			**0.002**			**0.004**
No	Ref.			Ref.		
Yes	4.5	(2.0–10.4)		4.5	(1.8–11.5)	
Any chemotherapy			**0.348**			**0.029**
No	Ref.			Ref.		
Yes	1.5	(0.6–3.5)		2.9	(1.0–8.1)	

*Abbreviations*: *CI* Confidence interval, *OR* Odds ratio, *N* Number, *P* P-value

^a^ Odds ratio from univariable logistic regression analysis

^b^*P*-values calculated from likelihood-ratio tests comparing survivors with and without chest wall abnormality

^c^ Odds ratio from multivariable logistic regression analysis, model adjusted for all factors shown

^d^ Including the following radiation fields: total body irradiation, mantle field, thorax, lungs, mediastinum, or thoracic spine

^e^ Including surgery to clavicle, scapulae and ribs, tumor excision from soft tissue on thorax, muscles on thorax, spine of thorax, and tumor or lymph node biopsy on the chest wall

### Telephone interviews

**Fig. 1:**
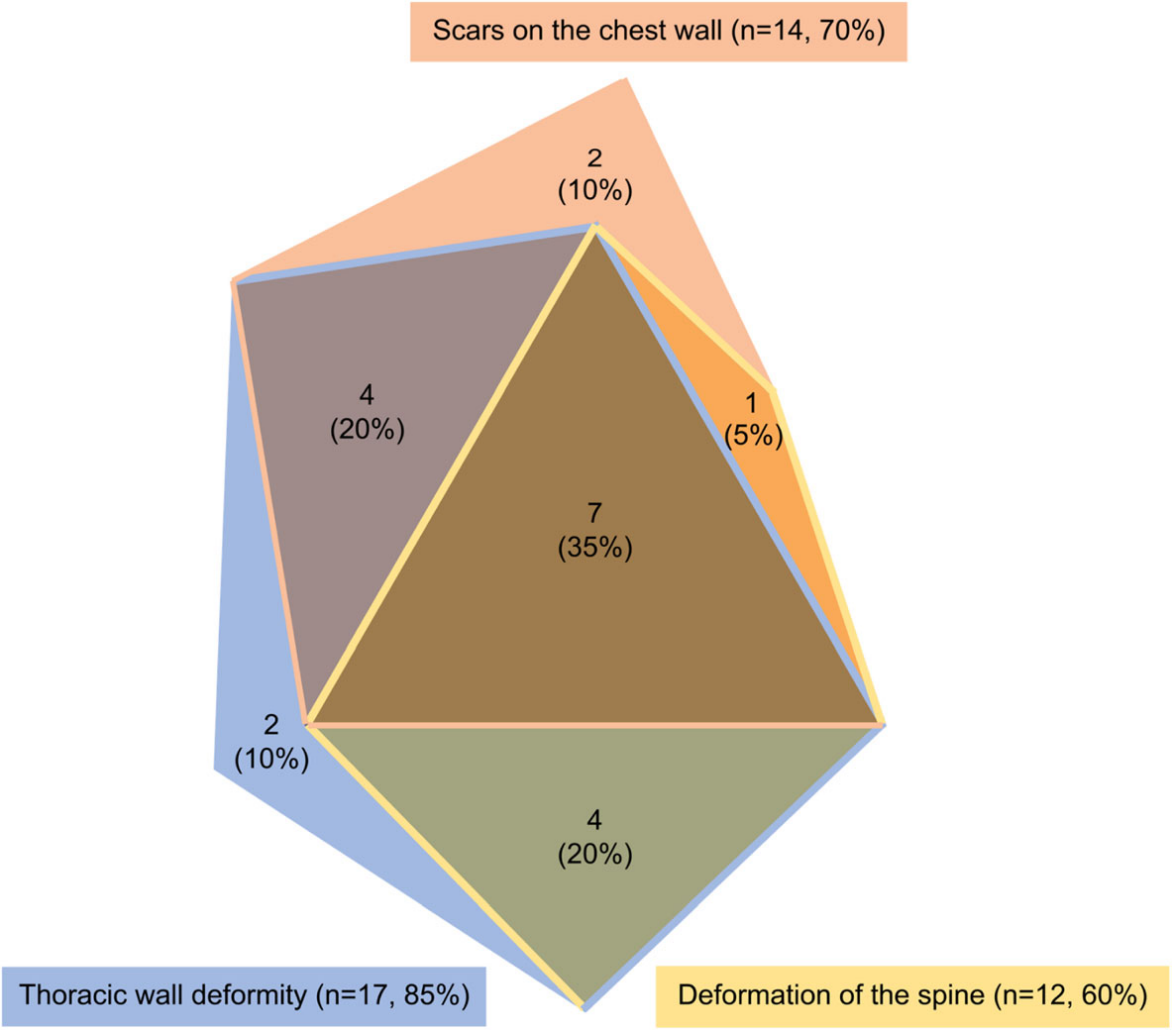
Proportional Venn diagram showing reported types and overlap of chest wall abnormalities in childhood cancer survivors in the telephone interview

Among the 48 survivors who reported chest wall abnormalities, 25 survivors were available for interview and 20 participated (80%) (Supplementary figure S1). Of the 20 interviewed, 18 were confirmed to have a chest wall abnormality (Table 3). When asked in more detail, 85% (17/20) described thoracic wall deformities, 60% (12/20) a deformation of the spine, and 70% (14/20) scars on the chest wall. Most survivors (80%; 16/20) reported multiple problems (Fig. 1 and Table 3). Thoracic wall deformities included pectus excavatum (*n* = 4), pectus carinatum (*n* = 2) and unspecified thoracic asymmetries (*n* = 6), missing or deformed ribs (*n* = 7), and deformation of the breasts (*n* = 1). Deformation of the spine included scoliosis (*n* = 7), hyper kyphosis (*n* = 4), and hyper lordosis (*n* = 3).

**Table 3:** Impact of chest wall abnormalities on the daily life of survivors, and medical attention required because of chest wall abnormality

	***N*** = 20	Proportion (%)
**Impact of chest wall abnormalities on daily life**		
Any impact	13	65%
Respiration	8	40%
Flexibility	7	35%
Activities of daily living	6	30%
Cosmetic	6	30%
Pain	6	30%
**Required medical attention because of chest wall abnormality**		
Any medical attention	15	75%
Consultation ^a^	14	70%
1 specialist visited	10	50%
> 1 specialist visited	4	20%
Diagnostic investigation ^b^	9	45%
Chest X-ray	6	30%
Chest X-ray + lung function test	3	15%
Operations	1	5%
Physiotherapy	6	30

*Abbreviations*: *N* Number; Respiration, any respiratory impairment; Flexibility, impairment of flexibility; Activities of daily living, inability or problems when performing activities of daily living such as housekeeping; Cosmetic, disturbed by the cosmetic appearance of the chest wall abnormality; Pain, any pain because of the chest wall abnormality; Consultation, ever consulted a medical doctor because of the chest wall abnormality; Diagnostic investigation, had further diagnostic testing because of the chest wall abnormality (e.g., chest x-ray, lung function tests); Operations, had an operation because of the chest wall abnormality; Physiotherapy, visited physical therapy because of the chest wall abnormalities

^a^ Survivors reported consultations with: general practitioner *n* = 5, pediatric oncologist *n* = 4, oncologist *n* = 1, orthopedist *n* = 3, chiropractor *n* = 1, surgeon *n* = 1, sports physician *n* = 1, rheumatologist *n* = 1

We also asked survivors whether the chest wall abnormalities affected their daily life and if they had sought medical attention. Among the 20 survivors interviewed, 13 survivors said that the chest wall abnormality affected respiration, flexibility, and activities of daily life, caused cosmetic problems, or pain (Table 3). Fifteen survivors had sought medical attention. Fourteen consulted a doctor (10 visited one specialist only and four visited multiple specialists). Specialists included general practitioners, pediatric oncologists, orthopedists, chiropractors, surgeons, sport physicians, or rheumatologists. Nine survivors underwent diagnostic testing, six had a chest x-ray only and three a chest x-ray and lung function tests. One survivor needed surgery and six received physiotherapy (Table 3).

### Literature review

Of the 2167 potentially relevant articles identified, we excluded 1935 articles after screening of title and abstracts, leaving 244 articles for full-text screening. Of those, we excluded a further 232 articles that did not meet the inclusion criteria (Supplementary figure S3). We summarize the remaining in Table 4. Among these, only two investigated an unselected cohort of survivors with regard to cancer diagnosis and treatment [11, 12]. Both used postal questionnaires (the Swiss and North American Childhood Cancer Survivor Studies) and found a prevalence of chest wall abnormalities of 2.0% [12] and 1.3% [11]. Six studies focused on survivors of selected cancer diagnoses [1–4, 9, 10]. Lucas et al. studied 23 survivors of chest wall sarcoma in the USA and found that one survivor (4%) had a ctCAE (Common Terminology Criteria for Adverse Events) Grade IV bone abnormality of the chest [1]. Utriainen et al. found that one of 21 (5%) Finnish survivors of high-risk neuroblastoma treated with hematopoietic stem cell transplantation had a sternal asymmetry [9]. Huang et al. found that, in the North American Childhood Cancer Survivor Study of 1653 survivors, 15 (0.4%) had a chest wall abnormality [10]. Perwein et al. studied 16 stage 4 neuroblastoma survivors and found that, of four (25%) with musculoskeletal late effects, one (6%) had an asymmetric pectus carinatum [2]. Tröbs et al. reported chest wall deformity in three (6%) of 49 German Wilms tumor survivors [3]. Heaston et al. studied 25 US Wilms tumor survivors and found radiographic evidence of abnormal skeletal development in 24 (96%) and hypoplasia of the pelvis and/or thorax in 13 (52%) [4].

**Table 4:** Literature summary of systematic review on chest wall abnormalities in childhood cancer survivors

First author, year, country	Treatment era	Inclusion criteria	Type of outcome assessment	Sample Size (n)	Age at diagnosis in years	Years of follow-up	Chest wall abnormality		
							Definition	n	%
**Study sample unselected with regard to cancer diagnosis and treatment**									
Kasteler 2017, CH [12]	1997–2005	Childhood cancer Survived 5 years from diagnosis ≥16 years old at survey	Postal questionnaire	1894	Median: 9 IQR: 4–14	Median: 18 IQR: 13–23	**Chest wall abnormalities**		
							- Ever in life	42	2.2%
							- After cancer diagnosis	38	2.0%
Mertens 2001, USA [11]	1970–1986	Childhood cancer Survived 5 years from diagnosis	Postal questionnaire	12,390	Range: 0–21	n.m.	**Chest wall abnormalities overall**	158	1.3%
							- Before diagnosis	22	0.2%
							- Diagnosis to end of treatment	39	0.3%
							- During first 5 years after end of treatment	21	0.2%
							- > 5 years after end of treatments	36	0.3%
**Studies focusing on selected diagnoses**									
Lucas 2017, USA [1]	10/06/ 2003–11/06/2011	Chest wall sarcoma survivors	CT or chest x-ray	23	Median: 12.5 Range: 3.6–20.6	Median: 9.25	**Bone abnormalities**		
							- ctCAE Grade IV (rib fracture with non-union resulting in pseudoarthrosis)	1	4%
Utriainen2017, FI [9]	1980–2000	High risk neuroblastoma survivors Treated with HSCT	Questionnaire, interview, hospital records, Physical examination	21	Median: 1.7 Range: 0.2–3.9	Median: 20 Range: 13–28	**Skeletal complications** - Sternal asymmetry	1	5%
Huang2013, USA [10]	1970–1986	CNS tumor survived 5 years from diagnosis	Postal questionnaire	1653	Median: 7.6 Range: 0–21	Median: 18.5 Range: 3.3–33.9	Kyphosis, scoliosis and/or acquired deformity of the chest or ribs	15	0.4%
Perwein 2011, A [2]	1984–09/2009	Stage 4 Neuroblastoma Regular presentation in follow-up program	Medical records review	16	Median: 1.8 Range: 0.2–10.7	Median: 4.3 Range: 0.4–23.2	**Musculoskeletal late effects**	4	25%
							- Asymmetric pectus carinatum	1	6.3%
Tröbs 2001, D ^3^	01/1974–12/1988	Wilms tumor	Medical records review	49	Median: 2.6	n.m	Chest wall deformity	3	6%
Heaston 1979, USA [4]	01/1954–12/1975	Wilms tumor Survived 4 years Megavoltage therapy Detailed medical data and serial radiographs available	Medical records review	25	Mean: 3.5 Range: 0.1–9.3	Mean: 9.8 Range: 4–18	**Axial Skeletal alternation (megavoltage)**		
							- Radiographic evidence of abnormal skeletal development	24	96%
							**Extra axial skeletal alterations**		
							- Hypoplasia of the pelvis and/or thorax	13	52%
**Studies focusing on specific cancer treatments**									
Venkatramani 2013, USA [8]	1999–2009	Childhood cancer Radiotherapy to the lungs without total body irradiation or whole lung irradiation	Medical records review	109	Median: 13.4 Range: 0.01–19.9	Median: 2.5 Range: 0.2–9	**Chest wall abnormality**	11	10%
							- Hypoplasia of chest wall	1	1%
Pintér 2003, HU [5]	01/01/1975–31/12/1983	Childhood cancer Operated for solid malign tumor excluding retinoblastomas and CNS tumors	Personal interviews, questionnaires	79	*N* = 17 < 1 year old *N* = 62 < 29 days old:	Mean: 20 Range: 16–25	Subgroup analysis per treatment group		
							**Surgical intervention (*n*** **= 15)**		
							- Thoracic deformity	4	27%
							- Scar formation resulting in psychological problems	12	80%
							**Multimodal therapy (surgical, chemo-, and radiotherapy,** ***n*** **= 64)**		
							- Skin lesion following radiotherapy	2	3%
							- Muscular deformity	7	11%
							- Bone morbidity (underdevelopment)	5	8%
							- Decreased physical ability to work	3	5%
							- Thoracic deformity	4	6%
							- Breast underdevelopment	7	11%
							- Scar formation resulting in psychological problems	12	19%
Butler 1990, USA [6]	1970–1987	Childhood cancer Survived to the age of skeletal maturity (14 years for females and 16 years for males) One year follow-up after irradiation Radiation to spine and/or extremities	Medical records review	143	Mean: 8.3 Range: 0.1–12.9	Mean: 9.9 Range: 2–18	**Chest and rib deformity**	51	36%
							In girls: breast asymmetry, none had treatment, although there were severe cosmetic deformities	7	35%
							**Significant pain at the radiation sites** (low back pain most common)	23	16.1%
Taylor 1997, UK [7]	1980–1986	Childhood Wilms tumor Abdominal radiotherapy Assessment of late effects through physician available	Follow-up forms filled by doctors	138	*N* = 50 < 3 years old *N* = 88 ≥ 3 years old	Median: 10.6	**Musculoskeletal late effects 8 of 27 described as ‘mild’**	27	19.6%
							- Asymmetry	13	9.4%
							- Hypoplasia	6	4.3%
							- Breast asymmetry	1	0.7%
							- Rib hypoplasia	1	0.7%

*Abbreviation*: *CNS* Central nervous system, *CT* Computer tomography, *ctCAE* Common terminology criteria for adverse events; *n.m* Not mentioned

Four studies focused on specific cancer treatments only [5–8]. Venkatramani et al. found that, of 109 US survivors treated with radiotherapy to the lungs without total body irradiation or whole lung irradiation, eleven (10%) had a chest wall abnormality—of those 11, one (1%) had hypoplasia of the chest wall [8]. Pintér et al. studied survivors who were operated on for solid malign tumors in Hungary [5]. In those with surgical treatment only (*n* = 15), four (27%) had a thoracic deformity and twelve (80%) had scar formation resulting in psychological problems. In those with multimodal therapy (*n* = 64), two (3%) had skin lesions following radiotherapy, seven (11%) had a muscular deformity, five (8%) had bone morbidity (underdevelopment), three (5%) had a decreased physical ability to work, four (6%) had a thorax deformity, seven (11%) had breast underdevelopment, and twelve (19%) had scar formation resulting in psychological problems. Butler et al. found that, of 143 US survivors 1 year after irradiation to the spine and/or extremities, 51 (36%) had a chest and rib deformity and 23 (16.%) had significant pain at the radiation sites [6]. Taylor et al. found that, out of 138 survivors of Wilms tumor after abdominal radiotherapy, 27 (19%) had musculoskeletal late effects, thirteen (9%) had asymmetry, six (4%) hypolplasia, one (0.7%) breast asymmetry, and one (0.7%) rib hypoplasia [7].

Studies that focused on selected outcomes or treatments reported a higher prevalence of chest wall abnormalities than studies based on unselected survivor cohorts (Table 4).

## Discussion

This is the first study to describe self-reported chest wall abnormalities in an unselected, representative sample of childhood cancer survivors in detail, and to validate answers in a structured interview. Two percent of all survivors reported a chest wall abnormality. We found a broad range of problems that were summarized as chest wall abnormalities. More than half of interviewed survivors were affected in their daily lives and three quarters required medical attention.

A strength of this study is that we clarified types of chest wall abnormalities and their impact on daily life by directly interviewing survivors. Survivors could explain their problems and we could inquire about the impact of their chest wall abnormalities. We were able to reach 80% of eligible survivors who had reported a chest wall abnormality in the SCCSS questionnaire. Participants did not differ from nonparticipants (results not shown) and a previous study concluded that response bias in the SCCSS did not markedly influence prevalence estimates [18]. A limitation of this study is that we were not able to validate chest wall abnormalities in survivors with medical exams or x-rays. A medical examination or chest x-ray would have helped to further quantify type and severity of the reported problems.

The prevalence of chest wall abnormalities in this study and in a previously published study from Switzerland with an overlapping population [12] was only slightly higher than in the North American Childhood Cancer Survivors Study (2.0% versus 1.3%) [11]. These two studies present data with similar methods (questionnaire survey) in an unselected cohort in respect to diagnoses and cancer treatment in either a national or multicenter set up. A further report of the North American Childhood Cancer survivors study on CNS tumor survivors found chest wall abnormalities in 0.4% of survivors—this is lower than in the group of CNS tumor survivors in our study (4%) [10]. Treatment-related factors like the frequency and cumulative dose of thoracic radiotherapy (such as spinal radiation in CNS tumor patients) might differ between countries and help to explain such differences in prevalence of chest wall abnormalities. Other studies and case series focusing on diagnostic subgroups mostly reported higher prevalence of chest wall abnormalities (4–52%) [1–4, 9]. They were all small, with 49 or less participants, and included high-risk tumor survivors who were exposed to intensive multimodal cancer treatment (stage 4 or high-risk neuroblastoma [2, 9]), survivors with tumors on the chest wall (chest wall sarcoma [1]), or diagnosed a long time ago, where radiotherapy and surgical approaches were far more invasive than today which in general sets survivors at higher risk of developing late effects [3, 4]. Four other studies focused on survivors of specific cancer treatments such as radiotherapy to the lungs [8], spine/extremities [6], or abdomen [7], or surgery of solid malign tumors [5] reported chest wall abnormalities in 1–36% of participants. In our study, those treated with thoracic radiotherapy or surgery to the chest had higher prevalence of chest wall abnormality than those without, reflecting the findings of the other studies.

Risk factors for chest wall abnormalities in survivors vary between studies. Our study is the first to report older age at diagnosis (16–20 years) as a risk factor for chest wall abnormalities. Peak bone growth velocity and increase of peak bone mass happen during puberty [19], therefore cancer treatment during this vulnerable time may affect the development of the spine and thoracic wall more severely than treatment earlier in childhood. CNS tumor survivors were most likely to report chest wall abnormalities. CNS tumors are often treated with radiotherapy to the spine, which is another risk factor identified in our study (OR 2.0; 95%CI 1.0–4.2), in the North American Childhood Cancer Survivor Study (rate ratio 5.0) [11], and in other studies [5–8]. Also, CNS tumor survivors often suffer from comorbidities that include small stature, functional deficits, endocrine diseases, fatigue, and psychological problems [20], which might lead to a higher subjective burden of chest wall abnormalities compared to other survivor groups.

We could validate chest wall abnormalities in 18 of 20 survivors who indicated a chest wall abnormality in the SCCSS questionnaire. Two survivors had scars only (which they reported as chest wall abnormalities) and 12 had both scars and chest wall abnormalities. This suggests that not all survivors understood the term “chest wall abnormality” as was intended by the questionnaire. We suggest that future questionnaires describe chest wall abnormalities in more detail or use open questions to further assess the type of chest wall abnormality. For physicians involved in follow-up care of childhood cancer survivors, awareness of chest wall abnormalities should be raised and clinical examinations performed to quantify the extent of individual problems and limitations.

Many participants were affected in daily life by chest wall abnormalities, which reflects the severity of this rare late effect after childhood cancer treatment. The most common complaints were impaired flexibility and physical fitness. An interdisciplinary treatment approach could help improve these issues. Early physiotherapy could be used in survivors at risk of developing chest wall abnormalities to improve late functional outcomes and might also reduce pain.

## Conclusion

In conclusion, this study suggests that, even though chest wall abnormalities are rare in the entire childhood cancer survivor population, they have a considerable impact on survivors’ lives. Physicians should pay close attention to these problems during follow-up care.

## Supplementary Information

**Additional file 1: Figure S1.** Study flowchart for the participation in the telephone interview on chest wall abnormalities in the Swiss Childhood Cancer Survivor Study. **Figure S2.** Original question in A) German, B) French) and C) English translation of original question for adults on pulmonary health in the SCCSS questionnaire. **Supplementary Text. Figure S3.** Prisma flow diagram of the article screening process.

**Additional file 2.**

**Additional file 3.**

## Data Availability

The datasets generated and/or analyzed during the current study are not publicly available due to the local data safety agreement but are available from the corresponding author on reasonable request.

## Abbreviations

CI: Confidence interval
CNS: Central nervous system
IQR: Interquartile range
N: Number
OR: Odds ratio
P: *P*-value
SCCR: Swiss Childhood Cancer Registry
SCCSS: Swiss Childhood Cancer Survivor Study
