# Supplementary material for: Validation of questionnaire-reported chest wall abnormalities with a telephone interview in Swiss childhood cancer survivors
Source: BMC Cancer. 2021 Jul 8;21:787. doi: 10.1186/s12885-021-08425-z (PMC8268220; doi:10.1186/s12885-021-08425-z)
Supplement: Supplementary file 2 — Additional file 2. [file 12885_2021_8425_MOESM2_ESM.pdf]

Fragebogen zu Veränderungen am Brustkorb, dem Brustbein und/oder an den Rippen

Datum:

____

Name und Geburtsdatum Survivor:

____

Name Interviewer:

____

Frage 1:

Haben Sie eine Veränderung am Brustkorb, an den Rippen oder am Brustbein?

□ **JA**

Falls JA, was für eine Veränderung/Veränderungen? Beschreiben Sie diese in Ihren eigenen Worten.

____

____

____

____

____

____

□ **NEIN**

Nun würden wir gerne einige Fragen zu häufig genannten Veränderungen stellen:

Frage 2:

Haben Sie Narben?

□ Falls **JA**, Wo: ____

____

Weiter mit Frage 2.1.

□ **NEIN,** weiter zu Frage 3

Frage 2.1.:

Ist dieses Problem vor oder nach der Krebserkrankung aufgetreten?

- □ vor der Krebsdiagnose
- □ während der Behandlung der Krebserkrankung
- □ nach der Behandlung der Krebserkrankung
- □ Ich weiss nicht mehr

Seit wann haben Sie diese Veränderung / dieses Problem ungefähr?

____

Frage 2.2.:

Haben sie deswegen irgendwelche Beschwerden?

□ **JA**, welche:

____

____

____

□ **NEIN**, überhaupt nicht

Frage 2.3.:

Stört es Sie kosmetisch / optisch?

□ **JA**: ____

____

____

□ **NEIN**

Frage 2.4.:

Haben sie auf Grund dieses Problems **Schmerzen**?

□ Falls **JA,** Wo: ______________________________

____

□ **NEIN**

Falls JA, wie häufig haben Sie Schmerzen?

____

Beeinträchtigen Sie diese Schmerzen im täglichen Leben?

□ NEIN

□ JA

Falls JA, Wie? ______________________________

____

Haben Sie auf Grund der Schmerzen schon **Schmerzmedikamente** eingenommen?

□ NEIN

□ JA

Haben Sie diese von einem Arzt verschrieben bekommen?

□ NEIN

□ JA

Was für ein Medikament? ______________________________

Wie oft nehmen dieses ein?

____

Frage 2.5.:

Haben Sie den Eindruck, dass sie wegen dem Problem / der Veränderung weniger beweglich sind?

□ Falls **JA**, bei was?

□ Probleme bei alltäglichen Tätigkeiten

____

□ Probleme beim Sport oder anderen körperlich fordernden Sachen:

____

□ **NEIN**

Frage 2.6.:

Waren Sie deswegen schon einmal beim Arzt oder haben Sie das Problem bei einem Arzt angesprochen?

□ Falls **JA**, Was für ein Arzt? □ **NEIN**

- □ Hausarzt
- □ Kinderonkologe
- □ Onkologe
- □ Hautarzt / Dermatologe
- □ Anderer ___________________________________

Wann? ____________________(Jahr)

Name und Adresse Arzt/Spital:

____

____

Frage 3:

Haben sie eine Veränderung der Form des Brustkorbes?

□ Falls **JA**:

Falls Ja, Was? ____

□ Asymmetrie des Brustkorbes:

□ Ist Ihr Brustbein nach innen eingezogen?

□ Anderes:

____

____

→ Weiter mit Frage 3.1.

□ **NEIN,** weiter mit Frage 4

Frage 3.1.:

Ist dieses Problem vor oder nach der Krebserkrankung aufgetreten?

□ vor der Krebsdiagnose

□ während der Behandlung der Krebserkrankung

□ nach der Behandlung der Krebserkrankung

□ Ich weiss nicht mehr

Seit wann haben Sie diese Veränderung / dieses Problem ungefähr?

____

Frage 3.2.:

Haben sie deswegen irgendwelche Beschwerden?

□ **JA**, welche:

____

____

____

□ **NEIN**, überhaupt nicht

Frage 3.3.:

Stört es Sie kosmetisch / optisch?

□ **JA**: ____

____

□ **NEIN**

Frage 3.4.:

Haben sie auf Grund dieses Problems **Schmerzen**?

□ Falls **JA,** Wo: ____

____

□ **NEIN**

Falls JA, wie häufig haben Sie Schmerzen?

____

Beeinträchtigen Sie diese Schmerzen im täglichen Leben?

□ NEIN

□ JA

Falls JA, Wie? ____

____

Haben Sie auf Grund der Schmerzen schon **Schmerzmedikamente** eingenommen?

□ NEIN

□ JA

Haben Sie diese von einem Arzt verschrieben bekommen?

□ NEIN

□ JA

Was für ein Medikament? ____

Wie oft nehmen dieses ein?

____

Frage 3.5.:

Sind Sie wegen des Problems körperlich eingeschränkt? z.B. Dass Sie im Alltag oder wenn sie Sport machen schneller müde werden oder gewisse Dinge nicht tun können.

□ Falls **JA**, bei was?

□ Probleme bei alltäglichen Tätigkeiten:

____

□ Probleme beim Sport oder anderen körperlich anstrengenden Sachen:

____

□ **NEIN**

Frage 3.6.:

Haben Sie das Gefühl, dass sie wegen des Problems manchmal Mühe mit der Atmung haben?

□ Falls **JA**, bei was?

□ Probleme bei alltäglichen Tätigkeiten

____

□ Probleme beim Sport oder anderen körperlich anstrengenden Sachen

____

□ **NEIN**

Frage 3.7.:

Haben Sie den Eindruck, dass sie wegen dem Problem / der Veränderung weniger beweglich sind?

□ Falls **JA**, bei was?

□ Probleme bei alltäglichen Tätigkeiten

____

□ Probleme beim Sport oder anderen körperlich fordernden Sachen:

____

□ **NEIN**

Frage 3.8.:

Waren Sie deswegen schon einmal beim Arzt oder haben Sie das Problem bei einem Arzt angesprochen?

□ Falls **JA**, Was für ein Arzt? □ **NEIN**

□ Hausarzt

□ Kinderonkologe

□ Onkologe

□ Orthopäde

□ Anderer ________________________________

Wann? __________________(Jahr)

Name und Adresse Arzt/Spital:

____

____

Frage 3.9.:

Haben sie auf Grund dieses Problems schon **Untersuchungen** gehabt?

□ Falls **JA,** □ **NEIN**

Welche?

- □ Röntgenbild des Brustkorbes
- □ Computertomographie des Brustkorbes
- □ Magnetresonanztomographie des Brustkorbes
- □ Lungenfunktionsmessung
- □ Andere: ____

Wann? _______________ (Jahr)

Name und Adresse Arzt/Spital:

____

____

Frage 3.10.:

Wurden sie auf Grund des genannten Problems bereits operiert oder ist eine Operation geplant?

□ **JA** □ **NEIN**

Was? ____

Wann? ____________________________ (Jahr)

Name und Adresse Arzt/Spital:

____

____

Frage 3.11.:

Waren Sie auf Grund dieses Problems in **Physiotherapeutischer** Behandlung?

□ **JA** □ **NEIN**, weiter zu Frage 20

Von wann bis wann? ________________-_____________(Jahre)

Welcher Arzt hat Ihnen die Physiotherapie verschrieben?

Name und Adresse Arzt/Spital:

____

____

Frage 4:

Haben Sie eine Veränderung der Wirbelsäule?

□ Falls **JA**, Was?

- □ Seitliche Verkrümmung der WS (Skoliose)
- □ Verkrümmung der WS nach hinten, ein sogenannter Rundrücken oder Buckel (Kyphose)
- □ Verstärktes hohles Kreuz (Lordose)
- □ Andere: ____________________

→ Weiter mit Frage 4.1.

□ **NEIN,** weiter mit Frage 5

Frage 4.1.:

Ist dieses Problem vor oder nach der Krebserkrankung aufgetreten?

- □ vor der Krebsdiagnose
- □ während der Behandlung der Krebserkrankung
- □ nach der Behandlung der Krebserkrankung
- □ Ich weiss nicht mehr

Seit wann haben Sie diese Veränderung / dieses Problem ungefähr?

____

Frage 4.2.:

Haben sie deswegen irgendwelche Beschwerden?

□ **JA**, welche:

____

____

____

□ **NEIN**, überhaupt nicht

Frage 4.3.:

Stört es Sie kosmetisch / optisch?

□ **JA**: ____________________

____

____

□ **NEIN**

Frage 4.4.:

Haben sie auf Grund dieses Problems **Schmerzen**?

□ Falls **JA,** Wo: ____

____

□ **NEIN**

Falls JA, wie häufig haben Sie Schmerzen?

____

Beeinträchtigen Sie diese Schmerzen im täglichen Leben?

- □ NEIN
- □ JA

Falls JA, Wie? ____

____

Haben Sie auf Grund der Schmerzen schon **Schmerzmedikamente** eingenommen?

- □ NEIN
- □ JA

Haben Sie diese von einem Arzt verschrieben bekommen?

- □ NEIN
- □ JA

Was für ein Medikament? ____

Wie oft nehmen dieses ein?

____

Frage 4.5.:

Sind Sie wegen des Problems körperlich eingeschränkt? z.B. Dass Sie im Alltag oder wenn sie Sport machen schneller müde werden oder gewisse Dinge nicht tun können.

□ Falls **JA**, bei was?

- □ Probleme bei alltäglichen Tätigkeiten

____

- □ Probleme beim Sport oder anderen körperlich fordernden Sachen:

____

□ **NEIN**

Frage 4.6.:

Haben Sie das Gefühl, dass sie wegen des Problems manchmal Mühe mit der Atmung haben?

□ Falls **JA**, bei was?

□ Probleme bei alltäglichen Tätigkeiten

____

□ Probleme beim Sport oder anderen körperlich anstrengenden Sachen

____

□ **NEIN**

Frage 4.7.:

Haben Sie den Eindruck, dass sie wegen dem Problem / der Veränderung weniger beweglich sind?

□ Falls **JA**, bei was?

□ Probleme bei alltäglichen Tätigkeiten

____

□ Probleme beim Sport oder anderen körperlich fordernden Sachen

____

□ **NEIN**

Frage 4.8.:

Waren Sie deswegen schon einmal beim Arzt oder haben Sie das Problem bei einem Arzt angesprochen?

□ Falls **JA**, Was für ein Arzt? □ **NEIN**

□ Hausarzt

□ Kinderonkologe

□ Onkologe

□ Orthopäde

□ Anderer ________________________________

Wann? ____________________(Jahr)

Name und Adresse Arzt/Spital:

____

____

Frage 4.9.:

Haben sie auf Grund dieses Problems schon **Untersuchungen** gehabt?

□ Falls **JA,** □ **NEIN**

Welche? ____

□ Röntgenbild des Brustkorbes

□ Computertomographie des Brustkorbes

□ Magnetresonanztomographie des Brustkorbes

□ Lungenfunktionsmessung

□ Andere: ____

Wann? ________________ (Jahr)

Name und Adresse Arzt/Spital:

____

____

Frage 4.10.:

Wurden sie auf Grund des genannten Problems bereits operiert oder ist eine Operation geplant?

□ **JA** □ **NEIN**

Was? ____

Wann? ______________________________ (Jahr)

Name und Adresse Arzt/Spital:

____

____

Frage 4.11.:

Waren Sie auf Grund dieses Problems in **Physiotherapeutischer** Behandlung?

□ **JA** □ **NEIN**, weiter zu Frage 20

Von wann bis wann? _________________-______________(Jahre)

Welcher Arzt hat Ihnen die Physiotherapie verschrieben?

Name und Adresse Arzt/Spital:

____

____

Frage 5:

Haben sie Veränderungen der Rippen?

□ Falls **JA**, Was?

□ teilweise oder ganz fehlende Rippen

□ Andere: ____

→ Weiter mit Frage 5.1.

□ **NEIN,** weiter mit Frage 6

Frage 5.1.:

Ist dieses Problem vor oder nach der Krebserkrankung aufgetreten?

□ vor der Krebsdiagnose

□ während der Behandlung der Krebserkrankung

□ nach der Behandlung der Krebserkrankung

□ Ich weiss nicht mehr

Seit wann haben Sie diese Veränderung / dieses Problem ungefähr?

____

Frage 5.2.:

Haben sie deswegen irgendwelche Beschwerden?

□ **JA**, welche:

____

____

____

____

□ **NEIN**, überhaupt nicht

Frage 5.3.:

Stört es Sie kosmetisch / optisch?

□ **JA**: ____

____

____

□ **NEIN**

Frage 5.4.:

Haben sie auf Grund dieses Problems **Schmerzen**?

□ Falls **JA,** Wo: ____

____

□ **NEIN**

Falls JA, wie häufig haben Sie Schmerzen?

____

Beeinträchtigen Sie diese Schmerzen im täglichen Leben?

□ NEIN

□ JA

Falls JA, Wie? ____

____

Haben Sie auf Grund der Schmerzen schon **Schmerzmedikamente** eingenommen?

□ NEIN

□ JA

Haben Sie diese von einem Arzt verschrieben bekommen?

□ NEIN

□ JA

Was für ein Medikament? ____

Wie oft nehmen dieses ein?

____

Frage 5.5.:

Sind Sie wegen des Problems körperlich eingeschränkt? z.B. Dass Sie im Alltag oder wenn sie Sport machen schneller müde werden oder gewisse Dinge nicht tun können.

□ Falls **JA**, bei was?

□ Probleme bei alltäglichen Tätigkeiten:

____

□ Probleme beim Sport oder anderen körperlich anstrengenden Sachen:

____

□ **NEIN**

Frage 5.6.:

Haben Sie das Gefühl, dass sie wegen des Problems manchmal Mühe mit der Atmung haben?

□ Falls **JA**, bei was?

- □ Probleme bei alltäglichen Tätigkeiten

  ____

- □ Probleme beim Sport oder anderen körperlich anstrengenden Sachen

  ____

□ **NEIN**

Frage 5.7.:

Haben Sie den Eindruck, dass sie wegen dem Problem / der Veränderung weniger beweglich sind?

□ Falls **JA**, bei was?

- □ Probleme bei alltäglichen Tätigkeiten

  ____

- □ Probleme beim Sport oder anderen körperlich fordernden Sachen:

  ____

□ **NEIN**

Frage 5.8:

Waren Sie deswegen schon einmal beim Arzt oder haben Sie das Problem bei einem Arzt angesprochen?

□ Falls **JA**, Was für ein Arzt? □ **NEIN**

- □ Hausarzt
- □ Kinderonkologe
- □ Onkologe
- □ Orthopäde
- □ Anderer ______________________________

Wann? ____________________(Jahr)

Name und Adresse Arzt/Spital:

____

____

Frage 5.9.:

Haben sie auf Grund dieses Problems schon **Untersuchungen** gehabt?

□ Falls **JA,** □ **NEIN**

Welche? ____

- □ Röntgenbild des Brustkorbes
- □ Computertomographie des Brustkorbes
- □ Magnetresonanztomographie des Brustkorbes
- □ Lungenfunktionsmessung
- □ Andere: ____

Wann? ________________ (Jahr)

Name und Adresse Arzt/Spital:

____

____

Frage 5.10.:

Wurden sie auf Grund des genannten Problems bereits operiert oder ist eine Operation geplant?

□ **JA** □ **NEIN**

Was? ____

Wann? ______________________________ (Jahr)

Name und Adresse Arzt/Spital:

____

____

Frage 5.11.:

Waren Sie auf Grund dieses Problems in **Physiotherapeutischer** Behandlung?

□ **JA** □ **NEIN**, weiter zu Frage 20

Von wann bis wann? ________________-______________(Jahre)

Welcher Arzt hat Ihnen die Physiotherapie verschrieben?

Name und Adresse Arzt/Spital:

____

____

Frage 6:

Veränderungen der Brust?

□ Falls **JA**, Was? ____

____

____

→ Weiter mit Frage 6.1.

□ **NEIN,** weiter mit Frage 7

Frage 6.1.:

Ist dieses Problem vor oder nach der Krebserkrankung aufgetreten?

- □ vor der Krebsdiagnose
- □ während der Behandlung der Krebserkrankung
- □ nach der Behandlung der Krebserkrankung
- □ Ich weiss nicht mehr

Seit wann haben Sie diese Veränderung / dieses Problem ungefähr?

____

Frage 6.2.:

Haben sie deswegen irgendwelche Beschwerden?

□ **JA**, welche:

____

____

____

____

□ **NEIN**, überhaupt nicht

Frage 6.3.:

Stört es Sie kosmetisch / optisch?

□ **JA**: ____

____

____

□ **NEIN**

Frage 6.4.:

Haben sie auf Grund dieses Problems **Schmerzen**?

□ Falls **JA,** Wo: ____

____

□ **NEIN**

Falls JA, wie häufig haben Sie Schmerzen?

____

Beeinträchtigen Sie diese Schmerzen im täglichen Leben?

- □ NEIN
- □ JA

Falls JA, Wie? ____

____

Haben Sie auf Grund der Schmerzen schon **Schmerzmedikamente** eingenommen?

- □ NEIN
- □ JA

Haben Sie diese von einem Arzt verschrieben bekommen?

- □ NEIN
- □ JA

Was für ein Medikament? ____

Wie oft nehmen dieses ein?

____

Frage 6.5.:

Haben Sie den Eindruck, dass sie wegen dem Problem / der Veränderung weniger beweglich sind?

□ Falls **JA**, bei was?

- □ Probleme bei alltäglichen Tätigkeiten

____

- □ Probleme beim Sport oder anderen körperlich fordernden Sachen:

____

□ **NEIN**

Frage 6.6.:

Waren Sie deswegen schon einmal beim Arzt oder haben Sie das Problem bei einem Arzt angesprochen?

□ Falls **JA**, Was für ein Arzt? □ **NEIN**

- □ Hausarzt
- □ Kinderonkologe
- □ Onkologe
- □ Gynäkologe (Frauenarzt)
- □ Anderer ___________________________________

Wann? ___________________(Jahr)

Name und Adresse Arzt/Spital:

____

____

Frage 6.7.:

Haben sie auf Grund dieses Problems schon **Untersuchungen** gehabt?

□ Falls **JA,** □ **NEIN**

Welche? ____

____

Wann? ________________ (Jahr)

Name und Adresse Arzt/Spital:

____

____

Frage 6.8.:

Wurden sie auf Grund des genannten Problems bereits operiert oder ist eine Operation geplant?

□ **JA** □ **NEIN**

Was? ____

Wann? _______________________________ (Jahr)

Name und Adresse Arzt/Spital:

____

____

Frage 7:

Haben Ihre Mutter, Ihr Vater oder Ihre Geschwister Veränderungen am Brustkasten und/oder an den Rippen?

□ **JA** □ **NEIN**

Mutter: ____

Vater: ____

Geschwister 1: ______________________________________

Geschwister 2: ______________________________________

Geschwister 3: ______________________________________

Frage 8:

Wir sind jetzt am Ende des Interviews angelangt.

Haben Sie noch etwas, worüber sie gerne sprechen würden oder eine Anmerkung?

□ **JA** □ **NEIN**

____

____

____

____

____

Kontakterlaubnis: Dürfen wir die von Ihnen genannten Ärzte / Spitäler kontaktieren und um die Unterlagen zu Ihrer Erkrankung bitten?

□ JA □ NEIN

Bitte unterschreiben Sie die beiliegende **schriftliche Einverständniserklärung** und schicken Sie diese mit dem Fragebogen an uns zurück.
Alle erhaltenen Informationen werden im Rahmen der ärztlichen Schweigepflicht absolut vertraulich behandelt.
