# Supplementary material for: Validation of questionnaire-reported chest wall abnormalities with a telephone interview in Swiss childhood cancer survivors
Source: BMC Cancer. 2021 Jul 8;21:787. doi: 10.1186/s12885-021-08425-z (PMC8268220; doi:10.1186/s12885-021-08425-z)
Supplement: Supplementary file 3 — Additional file 3. [file 12885_2021_8425_MOESM3_ESM.pdf]

Questionnaire on alterations of the chest, sternum and/or rib cage

Date:

____

Survivor name and date of birth:

____

Name of the interviewer:

____

Question 1:

Have you noticed any alteration of your chest, ribs, or sternum?

□ **YES**

If **YES,** what kind of alteration? Please describe in your own words.

____

____

____

____

____

____

□ **NO**

We would like to ask you a couple of questions concerning frequently reported alterations/problems:

Question 2:

Do you have any visible scars?

□ If **YES**, where: ____

____

Continue with question 2.1.

□ **NO,** continue with question 3

Question 2.1.:

Did this alteration occur before or after you suffered from cancer?

- □ Previous to the diagnosis
- □ During cancer treatment
- □ After completion of the treatment
- □ I cannot remember

Since when have you had this alteration/problem?

____

Question 2.2.:

Does the described alteration cause you any discomfort?

□ **YES**, what kind:

____

____

____

□ **NO**, not at all.

Question 2.3.:

Does the visual aspect of it bother/upset you in any way?

□ **YES**: ____

____

____

□ **NO**

Question 2.4.:

Does the alteration cause you any physical pain/discomfort?

□ If **YES,** where: ____

____

□ **NO**

If **YES,** how frequently do you suffer from pain?

____

Does the pain impair you in your everyday life?

- □ NO
- □ YES

If yes, in what way? ____

____

Have you ever had to take painkillers because of the pain caused by the alteration?

- □ NO
- □ YES

Were those painkillers prescribed by a physician?

- □ NO
- □ YES

What type of painkiller did/do you take? ______________________________

How often do you have to take a painkiller?

____

Question 2.5.:

Do you have the impression, that the alteration impairs your flexibility/joint mobility?

□ If **YES**, during which kind of activity?

- □ Problems during everyday activities

____

- □ Problems during sports or other physical activities:

____

□ **NO**

Question 2.6.:

Has the alteration ever led you to consult a physician or have you ever talked about it with a physician during a consultation?

□ If **YES**, what type of physician? □ **NO**

- □ Family doctor
- □ Paediatric oncologist
- □ Oncologist
- □ Dermatologist
- □ Other ___________________________________

When? ____________________(year)

Name of the physician/clinic: ____

Address: ____

Question 3:

Have you noticed any alteration in the shape of your chest-wall?

□ If **YES**:

What kind of alteration? ____

□ Asymmetric chest-wall:

□ Does your sternum have a concave (caved-in) appearance? (funnel chest)

□ Other:

____

____

→ Continue with question 3.1.

□ **NO,** continue with question 4

Question 3.1.:

Did this alteration occur before or after you suffered from cancer?

□ Previous to the diagnosis

□ During cancer treatment

□ After completion of the treatment

□ I cannot remember

Since when do you have this alteration/problem?

____

Question 3.2.:

Does the described alteration cause you any discomfort?

□ **YES**, of which nature:

____

____

____

□ **NO**, not at all

Question 3.3.:

Does the visual aspect of it bother/upset you in any way?

□ **YES**: ____

____

____

□ **NO**

Question 3.4.:

Does the alteration cause you any physical pain/discomfort?

□ If **YES,** where: ____

____

□ **NO**

If **YES,** how frequently do you suffer from pain?

____

Does the pain impair you in your everyday life?

□ NO

□ YES

If yes, in what way? ____

____

Have you ever had to take painkillers because of the pain caused by the alteration?

□ NO

□ YES

Were those painkillers prescribed by a physician?

□ NO

□ YES

What type of painkiller did/do you take? ______________________________

How often do you have to take a painkiller?

____

Question 3.5.:

Are you physically impaired because of this alteration? Do you for example have the impression, that you are easily exhausted when performing everyday activities or physical activities or are you unable to perform certain activities?

□ If **YES**, which kind of activity?

□ Problems during everyday activities

____

□ Problems during sports or other physical activities:

____

□ **NO**

Question 3.6.:

Do you have the impression, that the alteration causes a shortness of breath during certain activities?

□ If **YES**, during which kind of activities?

□ Problems during everyday activities

____

□ Problems during sports or other physical activities

____

□ **NO**

Question 3.7.:

Do you have the impression, that the alteration impairs your flexibility/joint mobility?

□ If **YES**, during which kind of activity?

□ Problems during everyday activities

____

□ Problems during sports or other physical activities:

____

□ **NO**

Question 3.8.:

Has the alteration ever led you to consult a physician or have you ever talked about it with a physician during a consultation?

□ If **YES**, what type of physician? □ **NO**

□ Family doctor

□ Paediatric oncologist

□ Oncologist

□ Dermatologist

□ Other ______________________________

When? ____________________(year)

Name and address of the physician/clinic:

____

____

Question 3.9.:

Have you ever had a medical examination because of the alteration?

□ If **YES,** □ **NO**

Which kind of medical examination?

- □ Chest x-ray
- □ CT-scan
- □ MRI (magnetic resonance imaging)
- □ Lung function testing
- □ Other: ____

When? _______________ (year)

Name and address of the physician/clinic:

____

____

Question 3.10.:

Have you ever had an operation or is an operation planned because of this alteration?

□ **YES** □ **NO**

What kind of operation? ____

When? _____________________________ (year)

Name of the physician/clinic: Name and address of the physician/clinic:

____

____

Question 3.11.:

Do you have physiotherapy because of this alteration/problem?

□ **YES** □ **NO**

During which time? ______________________________(year)

Which physician prescribed the physiotherapy?

Name of the physician/clinic: Name and address of the physician/clinic:

____

____

Question 4:

Have you noticed an alteration of your spine?

□ If **YES**, what kind of alteration?

- □ Lateral distortion/ sideways curve of the spine (scoliosis)
- □ Abnormally pronounced convex curvature of the spine (kyphosis/hunchback)
- □ Abnormally pronounced inward/concave curving of the spine (lordosis)
- □ Other: ____
- → Continue with question 4.1.

□ **NO**, continue with question 5

Question 4.1.:

Did this alteration occur before or after you suffered from cancer?

- □ Previous to the diagnosis
- □ during cancer treatment
- □ after completion of the treatment
- □ I cannot remember

Since when do you have this alteration/problem?

____

Question 4.2.:

Does the described alteration cause you any discomfort?

□ **YES**, of which nature:

____

____

____

□ **NO**, not at all.

Question 4.3.:

Does the visual aspect of it bother/upset you in any way?

□ **YES**: ____

____

____

□ **NO**

Question 4.4.:

Does the alteration cause you any physical pain/discomfort?

□ If **YES,** where: ____

____

□ **NO**

If **YES,** how frequently do you suffer from pain?

____

Does the pain impair you in your everyday life?

- □ NO
- □ YES

If yes, in what way? ____

____

Have you ever had to take painkillers because of the pain caused by the alteration?

- □ NO
- □ YES

Were those painkillers prescribed by a physician?

- □ NO
- □ YES

What type of painkiller did/do you take? ____

How often do you have to take a painkiller?

____

Question 4.5.:

Are you physically impaired because of this alteration? Do you for example have the impression, that you are easily exhausted when performing everyday activities or physical activities or are you unable to perform certain activities?

□ If **YES**, which kind of activity?

□ Problems during everyday activities

____

□ Problems during sports or other physical activities:

____

□ **NO**

Question 4.6.:

Do you have the impression, that the alteration causes a shortness of breath during certain activities?

□ If **YES**, during which kind of activities?

□ Problems during everyday activities

____

□ Problems during sports or other physical activities

____

□ **NO**

Question 4.7.:

Do you have the impression, that the alteration impairs your flexibility/joint mobility?

□ If **YES**, during which kind of activity?

□ Problems during everyday activities

____

□ Problems during sports or other physical activities:

____

□ **NO**

Question 4.8.:

Has the alteration ever led you to consult a physician or have you ever talked about it with a physician during a consultation?

□ If **YES**, what type of physician? □ **NO**

□ Family doctor

□ Paediatric oncologist

□ Oncologist

□ Dermatologist

□ Other ______________________________

When? ____________________(year)

Name and address of the physician/clinic:

____

____

Question 4.9.:

Have you ever had a medical examination because of the alteration?

□ If **YES,** □ **NO**

Which kind of medical examination?

- □ Chest x-ray
- □ CT-scan
- □ MRT (magnetic resonance tomography)
- □ Lung function testing
- □ Other: ____

When? ________________ (year)

Name and address of the physician/clinic:

____

____

Question 4.10.:

Have you ever had an operation or is an operation planned because of this alteration?

□ **YES** □ **NO**

What kind of operation? ____

When? ______________________________ (year)

Name and address of the physician/clinic:

____

____

Question 4.11.:

Do you have physiotherapy because of this alteration/problem?

□ **YES** □ **NO**

During which time? _______________________________(year)

Which physician prescribed the physiotherapy?

Name and address of the physician/clinic:

____

____

Question 5:

Have you noticed an alteration of your ribs?

□ If **YES**, what kind of alteration?

□ partially of completely missing ribs

□ Other: ____

→ Continue with question 5.1.

□ **NO**, continue with question 6

Question 5.1.:

Did this alteration occur before or after you suffered from cancer?

□ Previous to the diagnosis

□ During cancer treatment

□ After completion of the treatment

□ I cannot remember

Since when have you had this alteration/problem?

____

Question 5.2.:

Does the described alteration cause you any discomfort?

□ **YES**, of which nature:

____

____

____

□ **NO**, not at all.

Question 5.3.:

Does the visual aspect of it bother/upset you in any way?

□ **YES**: ____

____

____

□ **NO**

Question 5.4.:

Does the alteration cause you any physical pain/discomfort?

□ If **YES,** where: ____

____

□ **NO**

If **YES,** how frequently do you suffer from pain?

____

Does the pain impair you in your everyday life?

□ NO

□ YES

If yes, in what way? ____

____

Have you ever had to take painkillers because of the pain caused by the alteration?

□ NO

□ YES

Were those painkillers prescribed by a physician?

□ NO

□ YES

What type of painkiller did/do you take? ______________________________

How often do you have to take a painkiller?

____

Question 5.5.:

Are you physically impaired because of this alteration? Do you for example have the impression, that you are easily exhausted when performing everyday activities or physical activities or are you unable to perform certain activities?

□ If **YES**, which kind of activity?

□ Problems during everyday activities

____

□ Problems during sports or other physical activities:

____

□ **NO**

Question 5.6.:

Do you have the impression, that the alteration causes a shortness of breath during certain activities?

□ If **YES**, during which kind of activities?

□ Problems during everyday activities

____

□ Problems during sports or other physical activities

____

□ **NO**

Question 5.7.:

Do you have the impression, that the alteration impairs your flexibility/joint mobility?

□ If **YES**, during which kind of activity?

□ Problems during everyday activities

____

□ Problems during sports or other physical activities:

____

□ **NO**

Question 5.8:

Has the alteration ever led you to consult a physician or have you ever talked about it with a physician during a consultation?

□ If **YES**, what type of physician? □ **NO**

□ Family doctor

□ Paediatric oncologist

□ Oncologist

□ Dermatologist

□ Other ___________________________________

When? ___________________(year)

Name and address of the physician/clinic:

____

____

Question 5.9.:

Have you ever had a medical examination because of the alteration?

□ If **YES,** □ **NO**

Which kind of medical examination?

- □ Chest x-ray
- □ CT-scan
- □ MRT (magnetic resonance tomography)
- □ Lung function testing
- □ Other: ____

When? _______________ (year)

Name and address of the physician/clinic:

____

____

Question 5.10.:

Have you ever had an operation or is an operation planned because of this alteration?

□ **YES** □ **NO**

What kind of operation? ____

When? ____________________________ (year)

Name and address of the physician/clinic:

____

____

Question 5.11.:

Do you have physiotherapy because of this alteration/problem?

□ **YES** □ **NO**

When? _____________________________(year)

Which physician prescribed the physiotherapy?

Name and address of the physician/clinic:

____

____

Question 6:

Have you noticed any alteration of your chest wall?

□ If **YES**, what kind of alteration? ____

____

____

→ Continue with question 6.1.

□ **NO**, continue with question 7

Question 6.1.:

Did this alteration occur before or after you suffered from cancer?

- □ Previous to the diagnosis
- □ During cancer treatment
- □ After completion of the treatment
- □ I cannot remember

Since when have you had this alteration/problem?

____

Question 6.2.:

Does the described alteration cause you any discomfort?

□ **YES**, of which nature:

____

____

____

□ **NO**, not at all.

Question 6.3.:

Does the visual aspect of it bother/upset you in any way?

□ **YES**: ____

____

____

□ **NO**

Question 6.4.:

Does the alteration cause you any physical pain/discomfort?

□ If **YES,** where: ____

____

□ **NO**

If **YES,** how frequently do you suffer from pain?

____

Does the pain impair you in your everyday life?

□ NO

□ YES

If yes, in what way? ____

____

Have you ever had to take painkillers because of the pain caused by the alteration?

□ NO

□ YES

Were those painkillers prescribed by a physician?

□ NO

□ YES

What type of painkiller did/do you take? ____

How often do you have to take a painkiller?

____

Question 6.5.:

Do you have the impression, that the alteration impairs your flexibility/joint mobility?

□ If **YES**, during which kind of activity?

□ Problems during everyday activities

____

□ Problems during sports or other physical activities:

____

□ **NO**

Question 6.6.:

Has the alteration ever led you to consult a physician or have you ever talked about it with a physician during a consultation?

□ If **YES**, what type of physician? □ **NO**

- □ Family doctor
- □ Paediatric oncologist
- □ Oncologist
- □ Dermatologist
- □ Other ___________________________________

When? ___________________(year)

Name and address of the physician/clinic:

____

____

Question 6.7.:

Have you ever had a medical examination because of the alteration?

□ If **YES,** □ **NO**

Which kind of medical examination? ____

____

When? _________________ (year)

Name and address of the physician/clinic:

____

____

Question 6.8.:

Have you ever had an operation or is an operation planned because of this alteration?

□ **YES** □ **NO**

What kind of operation? ____

When? ________________________________ (year)

Name and address of the physician/clinic:

____

____

Question 7:

Does anyone in your family have an alteration of their chest wall and/or their ribs?

□ **YES** □ **NO**

Mother: ____

Father: ____

Sibling 1: ____

Sibling 2: ____

Sibling 3: ____

Question 8:

We are now at the end of this interview.

Is there anything else you would like to discuss or a remark you would like to make?

□ **YES** □ **NO**

____

____

____

____

____

Permission to view medical reports: Do we have your permission to contact the physicians/clinics mentioned above and ask them to send us your medical reports?

□ YES □ NO

Please sign the written consent form in the attachment and return it with the questionnaire. All information received is strictly treated within the framework of medical confidentiality.
